# VariBench, new variation benchmark categories and data sets

**DOI:** 10.3389/fbinf.2023.1248732

**Published:** 2023-09-19

**Authors:** Niloofar Shirvanizadeh, Mauno Vihinen

**Affiliations:** Department of Experimental Medical Science, Lund University, Lund, Sweden

**Keywords:** variation, mutation, benchmark, method performance assessment, data sets, variation database

## 1 Introduction

Genetic variation data is nowadays easy to generate. Variation interpretation means the description of the significance of variations, often in relation to disease. This is substantially more difficult a problem than sequence generation. Experimental methods provide verified interpretations; however, due to huge amounts of variations in every individual, computational approaches are widely used. The length of human genome is over 3 billion base pairs (Nurk et al., 2022). Due to individual genetic heterogeneity, 4.1–5.0 million sites differ from the reference genome (Auton et al., 2015). Various types of prediction methods are widely used to interpret the variations, see (Niroula and Vihinen, 2016). Benchmark studies have indicated large differences in the performance of methods developed for the same type of variation prediction tasks, see e.g., (Thusberg et al., 2011; Niroula and Vihinen, 2019; Zhang et al., 2019; Marabotti et al., 2021; Anderson and Lassmann, 2022). Both predictor development and performance assessment are largely dependent on high-quality data. One might think that there is a large number of verified variations as the genetic diagnosis is widely applied; however, that is not the case, especially when considering specific types of variations or mechanisms.

The development and testing of computational methods are dependent on experimental data. Accurate prediction methods can be developed only with reliable experimentally verified cases with a systematic approach and using relevant measures (Vihinen, 2012; Vihinen, 2013). Method performance has to be assessed in comparison to existing knowledge. For that purpose, benchmark data sets with known and verified outcomes are needed. Such data sets can be time-consuming and costly to collect and require many manual steps. Therefore, it is important that the produced data are distributed and reused.

In the variation interpretation field, two databases deliver such data sets. VariBench (Nair et al., 2013; Sarkar et al., 2020) and VariSNP (Schaafsma et al., 2015) contain variation benchmark data. VariSNP is a version of the dbSNP database (Sherry et al., 2001) for short variations from where known disease-causing variants have been filtered away. VariBench is a generic database that contains all types of variations with all kinds of effects. These resources have been widely used for prediction method training and testing.

What requirements and criteria should benchmark data sets fulfill in relation to variation interpretation and in general? We have defined five criteria, discussed in (Nair et al., 2013). They include relevance, representativeness, non-redundancy, inclusion of both positive and negative cases and reusability. VariBench subscribes to the criteria and collects data sets and distributes them freely. VariBench data sets are frequently used to train and test method performance. These sets facilitate also post-publication comparison of methods to published benchmarks (Sarkar et al., 2020).

The bottleneck in sequencing projects has shifted from sequencing to interpretation of obtained results. Experimental studies of variant effects are the gold standard approaches. They are not feasible in many instances and therefore, various computational approaches have been developed. We divide the prediction methods into five categories in VariBench.

First, pathogenicity, also called tolerance, predictions aim to identify disease-related alterations of various types (for details see Table 1).These methods aim just to detect harmful or disease-related variants. Second, effect-specific methods are for the prediction of various effects at DNA, RNA and protein levels. Third, there are also predictors specific for certain molecules or families of molecules, typically for proteins. Fourth, some methods are dedicated to certain diseases. Fifth, some tools predict the phenotype, typically the severity of the variant effect.

**TABLE 1:** Types of data sets in VariBench.

Data set	Data sets in previous version	New data sets
Variation type data sets		
*Insertions and deletions*	4	2
*Substitutions coding region*		
Training data sets	23	9
Test data sets	5	3
*Structure mapped variations*		
General structural data sets	2	3
Transmembrane protein data sets	0	4
*Synonymous and unsense variants*	2	5
*Benign variants*	2	0
*Structural variants*	0	1
Effect specific data sets		
*DNA regulatory elements*	7	4
*RNA splicing*	15	6
*Protein aggregation*	2	0
*Binding free energy*	2	1
*Protein disorder*	1	1
*Protein solubility*	1	1
*Protein stability*	31	9
Single variants	21	9
Double variants	1	0
*Protein folding rate*	0	5
*Protein binding affinity*		
Generic protein-protein interactions	1	13
Antibody-antigen affinity changes	0	5
Protein-nucleic acid interactions	0	7
*Functional effects*		
Gain of function variants	0	1
Deep mutational data sets	0	7
Molecule-specific data sets	18	7
Disease-specific data sets		
*Cancer variation data sets*	4	4
*Other diseases*	8	2
Phenotype data sets	1	1

High-quality variation data sets are difficult and laborious to generate. VariBench collects, organizes, and integrates additional information and distributes different types of variation data sets. It is a unique database. We have updated the resource with 143 new data sets, which include more than 90 million variants. During the update, some new categories of variations and effects have been included. There are currently variations in 5 main categories, 17 subgroups and 11 groups.

## 2 Data sets and quality

VariBench collects from literature, databases and predictors data sets, which have been used to train methods or assess their performance. There are no selection criteria for the inclusion of data sets. This is because of several reasons. The data sets can be used as such, or they can be further cleaned and pruned to use in additional tasks, be extended with new cases, etc. A good benchmark data set should fulfill several requirements (Vihinen, 2012; Vihinen, 2013), including good coverage, representativeness and containing both positive and negative cases that are experimentally determined. The representativeness of amino acid substitution data sets was investigated (Schaafsma and Vihinen, 2018) and found not to be optimal.

The quality of data sets in VariBench is variable. We include even known low-quality data sets, since they may be valuable when building new data sets and for other applications. We have performed some quality tests, including consistency; however, it is the duty of the users of the data to evaluate whether the data are suitable for intended use. One of the goals of VariBench is to provide existing data sets, even when problematic, e.g., for comparative purposes.

Systematics is an integral part of data and database quality. It is quite common that due to errors and lack of systematics, all variants in an existing data set cannot be reused as they cannot be mapped to reference sequences.

An example of the importance of data quality is in the field of protein stability predictions. Most of the existing predictors are based on a single database, ProTherm, which was shown to contain numerous problems (Yang et al., 2018). Recently, new and higher-quality databases have emerged in this field (Stourac et al., 2021; Turina et al., 2021).

## 3 Uses of VariBench data

VariBench data sets have been widely used especially to train and test variation interpretation predictors (pathogenicity/tolerance, protein stability, solubility, melting temperature, gene/protein/disease-specific predictors, and interaction and structural effects on folded and disordered regions and proteins), but also in the benchmarking performance of tools for various types and effects. In addition to human, plant and animal-related predictors and benchmarks have benefitted from VariBench (Yang et al., 2022). The data has also facilitated the interpretation of variants according to the guidelines of American College of Medical Genetics and Genomics, and the Association for Molecular Pathology (ACMG/AMP) (Richards et al., 2015) and benchmarking such annotations.

## 4 Data sets in VariBench

VariBench contains now 559 files for separate data sets from 295 studies and covers a wide range of variations (Tables 1, 2). The data sets were collected from literature, websites and databases. They have been used for predictive purposes, most often to develop novel predictors for different types or effects of variants. Some data sets have been specifically collected for benchmarking purposes.

**TABLE 2:** New data sets in VariBench.

Origin of data^a^	Dataset first used for	Number of variants in each dataset	Number of different genes, transcripts or proteins in each dataset	References
Variation type datasets				
*Insertions and deletions*				
HGMD, gnomAD	MutPredIndel	231963, 4679, 1203	3556, 4679, 802	Pagel et al. (2019)
HGMD, gnomAD	MutPredLof	98095, 8840	13648, 1239	Pagel et al. (2019)
*Substitutions, coding region*				
Training datasets				
VariBench	PON-All	45573, 306, 5360, 324, 3836, 1109, 48176, 4154	14765, 232, 1261, 233, 704, 287, 13383, 1149	Yang et al. (2022)
HumDiv, HumVar, MGI, Disease Ontology Database, OMIA, UniProtKB, Ensembl	Mammalian diseases	377, 207, 62	131, 315, 51	Plekhanova et al. (2019)
http://www.arabidopsis.org, UniProt/Swiss-Prot, Ensembl	*Arabidopsis thaliana*	13707	999	Kono et al. (2018)
UniProt, SwissProt	Arabidopsis	4410	994	Kovalev et al. (2018)
HGMD, SwissVar, dbSNP	MutPred2	20643		Pejaver et al. (2020)
ClinVar, UniProt	DeepSav	43000, 43000	3386, 10974	Pei et al. (2020)
dbNSFP, ClinVar, HumsaVar, HGMD	VARITY	157708, 157708	3912, 3912	Wu et al. (2021)
ClinVar, gnomAD	MutScore	66037		Quinodoz et al. (2022)
HGMD, gnomAD	MutFormer	69159160		Jiang et al. (2021)
Test datasets				
ClinVar, HGMD, OMIM, gnomAD	Benchmarking with clinical data set	1757		Gunning et al. (2021)
ClinVar, VariBench	Benchmarking study	35167, 29173	3349, 8562	Anderson and Lassmann (2022)
ClinVar	Rett syndrome benchmark	4354	3217	Ganakammal and Alexov (2019)
*Structure mapped variants*				
General structural datasets				
ClinVar, ExAC, HumsaVar	Missense3D	1965, 2134		Ittisoponpisan et al. (2019)
UniProt	Protein structural analysis	6025, 4536	3782, 8211	Gao et al. (2015)
HumsaVar	Solvent accessibility	10760, 69385	1283, 12494	Savojardo et al. (2020)
Transmembrane proteins				
VariBench, ExAC	Transmembrane protein analysis	2058, 5422, 508, 1289, 1289	870, 5422, 508, 1289, 1289	Orioli and Vihinen (2019)
PDB	mCSM-membrane	347, 138/38, 16		Pires et al. (2020)
ClinVar, gnomAD	TMSNP	2624, 196 705		Garcia-Recio et al. (2021)
BorodaTM, PredMutHTP, TMSNP	MutTMPredictor	21379, 10031, 3706, 7374, 546	3341, 2114, 1183, 1848, 62	Ge et al. (2021)
*Synonymous and unsense variations*				
1KGP	Silva	33		Buske et al. (2013)
Silva, OMIM	TraP	75	376, 96, 102	Gelfman et al. (2017)
HGMD, dbDSM	usDSM	239358, 2400, 4502, 665, 5085		Tang et al. (2021)
ClinVar	Ensemble predictor	243, 243		Ganakammal and Alexov (2020)
1KGP, ExAC, gnomAD, generated data	Predictor review	1048576		Zeng and Bromberg (2019)
*Structural variations*				
ClinVar, gnomAD, ape sequences, 1KGP	StrVCTVRE	7669	5119	Sharo et al. (2022)
Effect-specific datasets				
*DNA regulatory elements*				
DNaseI-seq, ChIP-seq data	deltaSVM	45		Lee et al. (2015)
dbSNP, ClinVar, OMIM	ncVarDB	7228, 722		Biggs et al. (2020)
PRVCS, 1KGP, GTEx, GWAS catalogue	regBase	108, 67635, 796, 60393, 21725, 3105, 102, 7513, 61170, 5023, 11436, 61170		Zhang et al. (2019)
HGMD, ClinVar, OregAnno, GWAS catalog	WEVar	2874, 29		Wang et al. (2021)
*RNA splicing*				
BIC	EX-SKIP and HOT-SKIP	74, 42		Raponi et al. (2011)
ClinVar, literature	SQUIRLS	8322		Danis et al. (2021)
ClinVar, literature, InSiGHT	Cancer gene analysis	12, 347, 18	3, 32, 13	Moles-Fernández et al. (2018)
HGMD, SpliceDisease, DBASS	scdbNSFP	2959, 45		Jian et al. (2014)
Experimental data	SPiCE	142, 163,90	2, 2, 9	Leman et al. (2018)
ClinVar	CADD-Splice	1688852, 14011296, 1688852, 14011296		Rentzsch et al. (2021)
*Binding free energy*				
Skempi, literature	SAAMBE	2041, 1327	81, 43	Petukh et al. (2016)
*Protein disorder*				
SwissProt, VariBench	IDRMutPred	3348, 559, 5794, 5027	321, 26, 2562, 2390	Zhou et al. (2020)
*Protein solubility*				
VariBench, literature	PON-Sol2	5666, 46, 662	66, 9, 34	Yang et al. (2021)
*Protein stability*				
Single variants				
ProTherm	PreTherMut	836, 2530		Tian et al. (2010)
ProTherm	iStable	3131		Chen et al. (2013)
Experimental data	CAGI frataxin benchmark	8		Strokach et al. (2021)
ProTherm	iStable2	1564, 1495, 759, 265, 363, 129		Chen et al. (2020)
VariBench, ProtTherm	Benchmarking study	1024		Marabotti et al. (2021)
ProTherm	Thermonet	3214, 3214, 3214, 1744, 1744, 1744	148, 148, 148, 127, 127, 127	Li et al. (2020)
ProTherm, literature	ACDC-NN	[2197, 2050, 2046, 2231, 2042, 2094, 2300, 1933, 2007, 2284] [268, 183, 415, 187, 230, 376, 178, 170, 545, 96] [183, 415, 187, 230, 376, 178, 170, 545, 96, 268] [5, 199, 21, 75, 7, 1, 33 ] [5, 1, 199, 21, 75, 7, 1, 33] [1013, 813, 924, 1080, 1157, 1296, 1219, 1235, 1180] [268, 176, 398, 65, 143, 164, 66, 25, 143, 9] [176, 398, 65, 143, 164, 66, 25, 143, 9, 198]	[104, 107, 105, 103, 103, 103, 107, 111, 109, 104] [15, 13, 12, 15, 14, 15, 14, 11, 10, 13] [13, 12, 15, 14, 15, 14, 11, 10, 13, 15] [1, 4, 2, 2, 2, 1, 2] [5, 1, 199, 21, 75, 7, 1, 33] [63, 60, 60, 55, 56, 65, 65, 69, 69] [16, 7, 11, 7, 9, 14, 8, 5, 8, 1] [7, 11, 7, 9, 14, 8, 5, 8, 1, 8]	Benevenuta et al. (2021)
ThermoMutDB, ProTherm, VariBench	Benchmarking study	352		Pancotti et al. (2022)
*Protein folding rate*				
Experimental data	Kinetic data	806		Naganathan and Muñoz (2010)
Literature, PFD, kineticDB	KD-FREEDOM	467	15, 4	Huang and Gromiha (2010)
PFD, kineticDB	Fora	467, 154		Huang and Gromiha (2012)
PFD, kineticDB, literature	FREEDOM	467		Huang (2014)
Literature	UnfoldingRaCe and FoldingRaCe	790, 16, 60	26, 10, 5	Chaudhary et al. (2015), Chaudhary et al. (2016)
*Protein interaction*				
Generic protein-protein interactions				
Literature	CC/PBSA	582, 592	9, 57	Benedix et al. (2009)
SKEMPI, literature	Protein-protein binding affinity	123, 242, 574,1844	5, 9, 29, 81	Li et al. (2014)
SKEMPI	MutaBind	1925		Li et al. (2016)
SKEMPI	BindProfX	1 402		Xiong et al. (2017)
DACUM, SKEMPI, literature	iSEE	1102		Geng et al. (2019)
SKEMPI, ABbind, PROXiMATE, dbMPIKT	mCSM-PPI2	4196, 378	319, 19	Rodrigues et al. (2019)
SKEMPI, literature	MutaBind2	4191, 1707	319, 19	Zhang et al. (2020)
SKEMPI, CAPRI	SSIPe	1470, 734, 888, 190, 152	319, 19	Huang et al. (2020)
SKEMPI	NetTree	645, 1131, 4947, 4169, 8338, 787	29, 112, 319, 319, 319, 21	Wang et al. (2020)
PROXiMATE	ProAffiMuSeq	1061, 112	104, 53	Jemimah et al. (2020)
ClinVar, ProTherm, SKEMP, literature	ELASPIC2	16189, 2563	14227, 2378	Strokach et al. (2019)
SKEMPI	mmCSM-PPI	1340, 595, 272	296, 68, 24	Rodrigues et al. (2021)
TCGA, ICGC	e-MutPath	59712		Li et al. (2021a)
Antibody-antigen affinity				
AB-Bind	mCSM-AB	558		Pires and Ascher (2016)
Literature	SiPMAB	212		Sulea et al. (2016)
Literature	Free energy perturbation method	200		Clark et al. (2019)
SiPMAB	Consensus predictor	46		Kurumida et al. (2020)
AB-BIND, PROXiMATE, SKEMPI	mCSM-AB2	1810		Myung et al. (2020)
Protein-nucleic acid interactions				
ProNIT	mCSM-NA	662	369	Pires and Ascher (2017)
ProNIT	SAMPDI	104	13	Peng et al. (2018)
ProNIT, dbAMEPNI	PremPDI	219	49	Zhang et al. (2018)
ENCODE, POSTAR2	DeepClip	81	32	Grønning et al. (2020)
dbAMEMPNI	iPNHOT	293	105	Zhu et al. (2020)
ProNIT, dbAMEMPNI	SAMPDI-3D	101, 463, 200, 419, 227	26, 30, 49, 96, 18	Li et al. (2021b)
PDB, literture	Nabe	2506	473	Liu et al. (2021)
*Functional effects*				
Gain of function data sets				
Literature	fuNCion	3794, 6930		Heyne et al. (2020)
Deep mutational data sets				
Literature	DeepSequence	712218	31	Riesselman et al. (2018)
Literature	fuNTRp	303, 75, 102, 286, 56		Miller et al. (2019)
Literature	Functional effects	183204		Reeb et al. (2020)
Literature	Deep mutational landscape	6357, 6357		Dunham and Beltrao (2021)
Literature	Benchmarking study	230033	10	Livesey and Marsh (2020)
Literature	LacI	102, 4303	1, 1	Miller et al. (2017)
Literature	Liver pyruvate kinase	126	1	Martin et al. (2020)
Molecule-specific data sets				
	CFTR-MetaPred	1899, 1210		Rychkova et al. (2017)
Literature	CYSMA	141		Sasorith et al. (2020)
SwissProt, BTKbase	KinMutRF	3689	459	Pons et al. (2016)
SwissVar, HumsaVar, Ensembl Variation, ClinVar	Cardiac sodium channel variants	1392	1	Tarnovskaya et al. (2020)
Literature	SCN9A variants	85	1	Toffano et al. (2020)
Literature	Troponin variants	136	1	Shakur et al. (2021)
Literature, ClinVar, HGMD	IDUA	147	1	Borges et al. (2021)
Disease-specific data sets				
*Cancer variation data sets*				
Literature	dbCID	57, 153, 728	22, 39, 46	Yue et al. (2019)
Literature	dbCPM	108, 863, 1109	11, 71, 130	Yue et al. (2018)
ICGC, TCGA, Pediatric Cancer Genome Project	MutaGene	5276	58	Goncearenco et al. (2017)
UMD_TP53, TP53MULTLOAD	TP53_PROF	1362, 1295	1, 1	Ben-Cohen et al. (2022)
*Other diseases*				
ClinVar, gnomAD, literature	CardioBoost	1237, 215, 154, 308, 532 218, 289, 2003,2578 218, 289, 2003, 2578 347, 463, 170 106, 106, 35 157, 227, 75 157, 227, 75	7, 6,6,7, 9 16, 16, 16, 21 16, 16, 16, 21 12, 8, 11 1, 1, 1 1, 1, 1 1, 1, 1	Zhang et al. (2021)
HGMD, dbSNP	Steroid metabolism diseases	797	12	Chan (2013)
COSMIC	Benchmarking cancer variants	164	11	Petrosino et al. (2021)
Phenotype data sets				
ClinVar	VusPrize	45749, 25080, 684, 4843, 51091	2106, 1615, 244, 1239, 2828	Mahecha et al. (2022)

^a^ Abbreviations: 1KGP, thousand genomes project; HGMD, human gene mutation database; ICGC, international cancer genome consortium; PDB, protein data bank; TCGA, the cancer genome atlas.

There are 247 new data files that contain total 90,886,959 variants. Together with previous versions, there are 105,181,219 variants, the increase is more than seven-fold from the original number of 14,294,260 variants. The number of data sets is high because many articles contain more than one data set. Many of the data sets are redundant as they contain data from the same origin. The most common sources of variants are ClinVar (Landrum et al., 2018) database of variants and their disease relationship, ProTherm thermodynamic database (Kumar et al., 2006), and VariBench itself. The number of unique variants is significantly lower than the sum of the variants in the data sets.

The data sets are divided into 5 categories, 17 subgroups and 11 groups (Table 1). The amount of data items varies for independent sets and is dependent on the original data. Data items irrelevant to VariBench (i.e., not describing variants or their effects) were removed when sets were included to the database. In many data sets, variants are described at three molecular levels (DNA, RNA and protein) and sometimes also at protein structural level. One of the aims of VariBench is to facilitate the reuse of existing data sets, therefore the data are provided in as many levels as possible. Further, the data can be used for various purposes, beyond the original application, such as benchmarking, developing different types of predictors, bioinformatics reviews and analyses of variation types, clinical variation interpretation, etc. When doing such an extension, the users must be cautious and aware of the possible limitations of the data sets and to understand how they have been collected.

The main categories of variation type data sets are insertions and deletions, substitutions in coding and non-coding regions, structure-mapped variants, synonymous and unsense variants, benign variants, and DNA structural variants (See Tables 1, 2). Unsense variants are a new category for exonic alterations that may look synonymous, but affect the protein or its expression, typically due to aberrant splicing or miRNA binding alterations (Vihinen, 2022; Vihinen, 2023a; Vihinen, 2023b). Effect-specific data sets include DNA regulatory elements, RNA splicing, and protein property for aggregation, binding free energy, disorder, solubility, stability, folding rate, interactions, and functional effects. Molecule- and disease-specific data sets include information for individual genes, proteins, gene/protein families or diseases. Phenotype data sets are for a disease feature, severity of the phenotype.

Almost all the categories contain new data sets. In addition, we have 6 new variation categories including structural variations in DNA (1 data set), protein folding rate (5 data sets in six publications), antibody-antigen affinity changes (5 articles and sets), protein-nucleic acid interactions (6 articles), gain of function variants (Nurk et al., 2022), and deep mutational data sets (7 studies).

One of the new categories is for functional effects under the effect-specific category. These sets are mainly for massively parallel reporter assays (saturation mutagenesis) experiments. Users of these data have to be careful since the included data sets display a measured effect; however, their relevance to biological effect is not always clear, see (Vihinen, 2021). The functional effect does not necessarily mean biological effect. One would likely say that a reduction of more than 50% of e.g., enzyme activity has a functional effect. There are several diseases where 90% or more of the normal activity has to be lost for an individual to have a disease and show the effect on biological activity (Vihinen, 2021). Examples include hemophilias due to factor II, VII, IX, X or XII variations and severe immunodeficiency caused by adenosine deaminase alterations.

## Data Availability

Publicly available datasets were analyzed in this study. This data can be found here: http://structure.bmc.lu.se/VariBench.
